# The association of COVID-19 pandemic with the increase of sinogenic and otogenic intracranial infections in children: a 10-year retrospective comparative single-center study

**DOI:** 10.1007/s10143-024-02442-9

**Published:** 2024-05-07

**Authors:** Mohammed Issa, Vasiliki Kalliri, Sara Euteneuer, Arne Krümpelmann, Angelika Seitz, Olaf Sommerburg, Jens H. Westhoff, Steffen Syrbe, Pavlina Lenga, Martin Grutza, Moritz Scherer, Jan-Oliver Neumann, Ingo Baumann, Andreas W. Unterberg, Ahmed El Damaty

**Affiliations:** 1 Faculty of Medicine, Heidelberg University, Heidelberg, Germany; 2 Department of Neurosurgery, Heidelberg University Hospital, Im Neuenheimer Feld 400, D-69120 Heidelberg, Germany; 3 Department of Otorhinolaryngology, Heidelberg University Hospital, Heidelberg, Germany; 4 Center for Pediatric and Adolescent Medicine, Department I, University Hospital Heidelberg, Heidelberg, Germany; 5 Deptartment of Neuroradiology, Heidelberg University Hospital, Heidelberg, Germany

**Keywords:** Intracranial infection, Sinogenic, Otogenic, Pediatric patients, COVID-19

## Abstract

Objective: Otitis media and sinusitis are common childhood infections, typically mild with good outcomes. Recent studies show a rise in intracranial abscess cases in children, raising concerns about a link to COVID-19. This study compares a decade of data on these cases before and after the pandemic. Methods: This retrospective comparative analysis includes pediatric patients diagnosed with otitis media and sinusitis, who later developed intracranial abscesses over the past decade. We collected comprehensive data on the number of cases, patient demographics, symptoms, treatment, and outcomes. Results: Between January 2013 and July 2023, our center identified 10 pediatric patients (median age 11.1years, range 2.2–18.0 years, 60% male) with intracranial abscesses from otitis media and sinusitis. Of these, 7 cases (70%, median age 9.7 years, range 2.2–18.0 years) occurred since the onset of the COVID-19 pandemic, while the remaining 3 cases (30%, median age 13.3 years, range 9.9–16.7 years) were treated before the pandemic. No significant differences were found in otolaryngological associations, surgical interventions, preoperative symptoms, lab findings, or postoperative antibiotics between the two groups. All patients showed positive long-term recovery. Conclusion: This study reveals 5-fold increase of pediatric otogenic and sinogenic intracranial abscess cases in the last three-years since the onset of the COVID-19 pandemic. While further investigation is needed, these findings raise important questions about potential connections between the pandemic and the severity of otitis media and sinusitis complications in children. Understanding these associations can improve pediatric healthcare management during infectious disease outbreaks.

## Introduction

Otitis media and sinusitis are common infections in pediatric patients, typically presenting with mild symptoms and following an uncomplicated course [4, 9]. However, in rare cases, these infections can give rise to severe complications in the form of otogenic and sinogenic intracranial infections. Such complications occur when the infection spreads from the middle ear or sinuses to adjacent structures within the skull, leading to the formation of abscesses containing infected material and pus. These abscesses can exert pressure on critical brain structures and blood vessels, potentially causing life-threatening conditions such as meningitis, cerebral edema, or venous sinus thrombosis [11, 12, 16, 28].

Given the rarity and seriousness of otogenic and sinogenic intracranial infections, prompt identification and aggressive treatment are imperative to prevent devastating outcomes, including surgical intervention to drain the abscess and administer broad spectrum empiric antibiotic therapy [6]. Early diagnosis and intervention are crucial to limit the spread of infection and minimize the risk of neurological deficits and long-term disabilities [6, 21].

Despite a significant decrease in pediatric acute otitis media and sinusitis cases during the COVID-19 pandemic [17, 19], recent studies have reported a concerning increase in the number of pediatric patients presenting with otogenic and sinogenic intracranial infections during this same period [1, 2]. Notably, these infections demonstrated rapid intracranial progression with pronounced symptoms and neurological deficits [2, 17].

In light of this trend, our retrospective study aims to offer valuable insights into otogenic and sinogenic intracranial infections in pediatric patients. Through examining the clinical cases over a decade, comparing data before and since onset of the COVID-19 pandemic, and analyzing patients’ characteristics, clinical and microbiological outcomes, as well as surgical and antibiotic therapies, we aim to contribute to the existing knowledge on this subject and identify potential associations with the recent pandemic. We hope to elucidate this problem and raise the awareness aiming to early detection and management of these severe complications in pediatric populations.

## Methods

### Study design and patient population

This retrospective evaluation comprises a consecutive clinical series of cases that underwent neurosurgical and otolaryngological evacuation for otogenic or sinogenic intracranial abscesses in pediatric population between January 2013 and July 2023. The patients were classified into two groups: the pre-COVID-19 group (2013–2019) and the after onset-COVID-19 pandemic group (2020 - June 2023). Pediatric patients with intracranial infections due to other causes, such as postoperative complications after craniotomy or trauma, were excluded from the study. Immunocompromised patients, who suffered intracranial infections as a result of their primary disease or therapy (e.g., chemotherapy for haemato-oncological conditions), were also excluded. The institutional ethics committee approved the study, and the need for informed patient consent was waived for this retrospective series (Ref S-489/2023).

### Outcome parameters

We evaluated various clinical parameters, including sex, age, otolaryngological site of infection, surgical interventions, preoperative symptoms, laboratory, and microbiological findings, as well as pre- and postoperative antibiotics therapy. Clinical outcomes were assessed through a comprehensive review of medical charts, analyzing the neurological status pre- and postoperatively, morbidity, and mortality during hospitalization and follow-up.

### Radiological outcome

Diagnoses of intracranial pathology were based on magnetic resonance imaging (MRI) and computed tomography (CT) scans. Routine clinical and radiological follow-up examinations were performed before patient discharge.

### Statistical analysis

Statistical analysis was conducted to compare the number of cases and demographic characteristics between the pre-COVID-19 and after onset-COVID-19 pandemic groups. Normal distribution was assessed using the Shapiro–Wilk test. Continuous variables were presented as median and range, and categorical variables as frequencies and percentages. A p-value less than 0.05 was considered statistically significant, determined by the t-test for continuous variables and chi-squared test for nominal variables. SPSS 27 (IBM-Corp, Armonk, NY, USA) was used for all statistical analyses.

## Results

### Incidence of infections among Pediatric patients before and during the pandemic

**Fig. 1:**
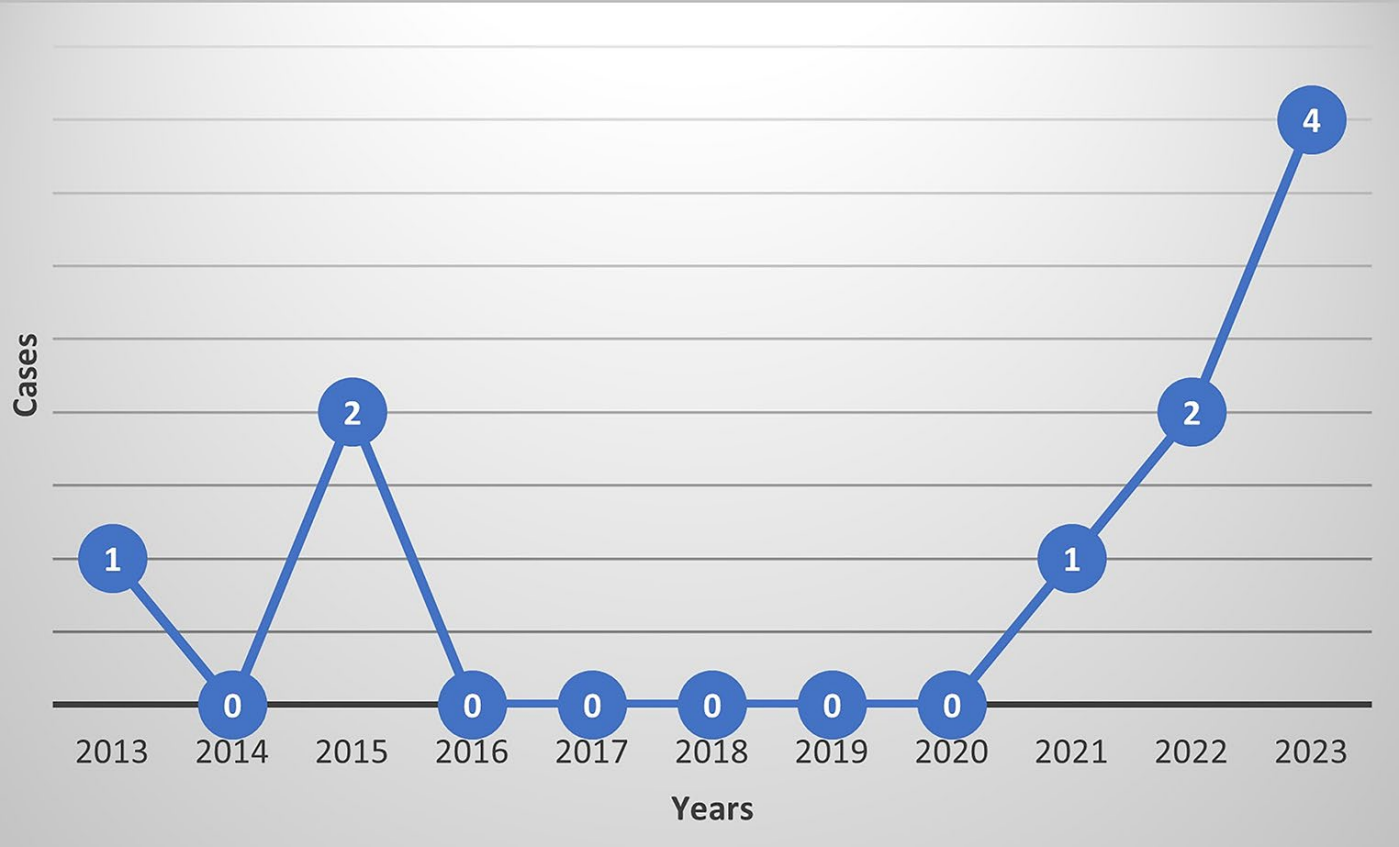
Incidence of intracranial infections due to otitis media or sinusitis over the last decade

Over a 10-year period, spanning from January 2013 to July 2023, a total of 10 cases of intracranial infections due to otitis media or sinusitis were reported in our center, meeting the inclusion criteria. As depicted in the diagram below (Fig. 1), only three patients experienced the disease from 2013 to 2019 (*N* = 3, 30%), resulting in an annual incidence of 0.43 case. However, since the onset of the pandemic, there has been a marked increase, with 7 cases reported between 2020 and July 2023 (*N* = 7, 70%), resulting in a higher incidence rate of 2 cases per year. Notably, during the years 2016 to 2020, there were no cases of intracranial infections of otogenic or sinogenic origin referred to our hospital.

### Demographic and clinical characteristics

The age distribution of the patients revealed no specific demographic characteristics (13.3 vs. 9.7 years of age in the pre- and during COVID-19 groups, respectively), as all were German inhabitants with no history of travel abroad before the onset of the disease. The pre-COVID-19 group consisted of all male patients, whereas the second group had a majority of female patients (*N* = 4, 57.1%). Only one patient in the entire cohort had a history of mild bronchial asthma, and there were no known predisposing diseases or immunodeficiency among the patients. In Table 1 all patients’ characteristics are summarized. The geographical regions covered through our center were the same in both groups.

**Table 1:** Cases characteristics, clinical and microbiological presentation

	Total	Pre-COVID-19	After-onset of COVID-19	*p*-value*
**Number of patients**	10	3 (30.0)	7 (70.0)	0.344
**Average of cases per a year**	1.0	0.43	2	**< 0.01**
**Sex**				
Male Female	6 (60.0) 4 (40.0)	3 (100.0) 0 (100.0)	3 (42.9) 4 (57.1)	0.167
**Median age in years (range)**	11.1 (2.2–18.0)	13.3 (9.9–16.7)	9.7 (2.2–18.0)	0.322
**Median Follow-up (range)**	8.7 (3.1–49.8)	39.6 (5.6–49.8)	7.9 (3.1–21.8)	**0.034**
**Otolaryngological site of infection**				
Sinusitis Otitis media	7 (70.0) 3 (30.0)	3 (100.0) 0 (0.0)	4 (57.1) 3 (42.9)	0.475
**Surgical interventions**				
Neurosurgical Otolaryngological	13 (54.2) 11 (45.8)	4 (30.8) 3 (27.3)	9 (69.2) 8 (72.7)	1.0 1.0
**Symptoms onset in days**	12 (0–75)	7 (0–14)	13 (0–75)	0.319
**Intracranial radiological findings**				
Epidural Subdural Epi- and subdural	6 (60) 3 (30) 1 (10)	1 (33.3) 2 (66.7) 0 (0.0)	5 (71.4) 1 (14.3) 1 (14.3)	0.524 1.0
**Recent antibiotics**	10 (90.0)	3 (100.0)	6 (85.7)	1.0
**Laboratory findings**				
CRP, mg/dl (range) WBC count, x 10^9^/L (range)	111.5 (3–311) 16.3 (6.7–27)	5 (3–311) 18.8 (6.7–19)	123 (41–230) 14.6 (7–27)	0.677 0.721
**Bacterial growth in cultivation**				
Prevotella loescheii Streptococcus intermedius Streptococcus pneumoniae Staphylococcus epidermidis	*7 (70.0)* 1 (10) 4 (40) 1 (10) 2 (20)	*2 (66.7)* 1 (33.3) 1 (33.3) 0 (0.0) 0 (0.0)	*5 (71.4)* 0 (0.0) 3 (42.9) 1 (14.3) 2 (28.6)	1.0 0.3 1.0 1.0 1.0
**Postoperative antibiotics**				
Metronidazole Ceftriaxone Cefotaxime Vancomycin Meropenem Rifampicin Ampicillin Teicoplanin Ceftadizim	9 (90) 5 (50) 3 (30) 4 (40) 5 (50) 2 (20) 2 (20) 1 (10) 1 (10)	3 (100.0) 1 (33.3) 1 (33.3) 1 (33.3) 1 (33.3) 2 (66.7) 0 (0.0) 0 (0.0) 0 (0.0)	6 (85.7) 4 (57.1) 2 (28.6) 3 (42.9) 4 (57.1) 0 (0.0) 2 (28.6) 1 (14.3) 1 (14.3)	1.0 1.0 1.0 1.0 1.0 1.0 1.0 1.0 1.0
**Change of Antibiotics**	4 (33.3)	0 (0.0)	4 (57.4)	0.2
**Postoperative improvement** **Remaining complaints**	10 (100.0) 2 (20.0)	3 (100.0) 1 (33.3)	7 (100.0) 1 (14.3)	1.0 1.0

(%) Data in parenthesis are percentages. *Bold denotes statistical significance

### Clinical symptoms, otolaryngological site of infection and radiological findings

The clinical symptoms before surgery were associated with the radiological findings of the anatomical localization of the infection (Table 2). Only one patient with seizures required immediate admission to the intensive care unit (ICU), while the remaining cases were admitted to the ICU only after surgery for postoperative monitoring. Before the pandemic, the primary cause of intracranial infections was sinusitis in all cases. All patients with otitis media were complicated with mastoiditis. Surprisingly, since the beginning of the COVID-19 pandemic, the cases were almost equally divided between otitis (42.9%) and sinusitis (57.1%) as the cause of intracranial infection, see Table 1.

**Table 2:** Location of intracranial abscess and their preoperative symptoms

Group	Preoperative Symptoms
**Before Covid**	
pansinusitis, subdural empyema over the whole left hemisphere	face swelling, right side hemiparesis, diplopia, facial nerve paresis, seizures
frontal sinusitis, subdural empyema of the right frontal region	fever, headache
frontal sinusitis, frontal epidural abscess	headache, face swelling
**After onset of Covid**	
otitis-mastoiditis, suboccipital epidural abscess	vomiting, swelling behind the ear
otitis-mastoiditis, temporal epidural abscess	fever, swelling behind the ear
otitis-mastoiditis, temporal epidural abscess	fever, ear pain, seizures
frontal sinusitis, frontal epidural abscess	fever, headache, vomiting, face swelling
pansinusitis, interhemispheric subdural empyema	fever, headache, mild hemiparesis on the right side
frontal and ethmoidal sinusitis, frontotemporal epidural abscess, and subdural empyema	headache, mild speech disorder
frontal sinusitis, frontal epidural abscess	Headache, ptosis, vomiting

In all cases, further investigation of the above-mentioned symptoms included brain imaging using magnetic resonance tomography (MRI). The findings regarding the localization of the infection and the number of surgeries required for treatment are summarized in Table 1.

In the pre-COVID-19 group, MRI revealed an epidural abscess in one patient (*N* = 1, 33%) and a subdural empyema in two patients (*N* = 2, 67%). One patient presented with an extensive subdural empyema, covering a significant portion of the left hemisphere, while the other two patients showed localized empyema in the frontal lobe, as expected from sinogenic origins.

Among the after-onset COVID-19 group, four patients exhibited an epidural abscess in MRI scans (*N* = 5, 71.4%), one patient had a subdural empyema (*N* = 1, 14.3%), and one patient had both epidural and subdural localizations of the infection. The patients with otogenic origin of intracranial infection showed temporal localization. One case demonstrated an infection in the interhemispheric fissure, originating from a widely spread pansinusitis. None of the patients in this group had a history of known COVID-19 infection or vaccination. Throughout their hospital stay, all seven patients tested negative for the coronavirus.

### Surgical treatment

In both groups, the majority of cases underwent only one neurosurgical and one otorhinolaryngological procedure, either during a single combined session or in separate sessions.

In the first group, before the COVID-19 pandemic, only one patient required a second neurosurgical operation to extend the previous craniotomy. This was necessary due to the significant expansion of the intracranial infection over the subdural frontotemporal region. Overall, the average number of surgeries was 1.3 for neurosurgical procedures and 1.1 for otorhinolaryngological procedures. The otorhinolaryngological procedure in these sinogenic-origin only cases consisted of endoscopic sinus surgery, targeting the primary source of the infection in the ethmoid and frontal sinuses. Neurosurgical procedures in the first group consisted of a frontal craniotomy in two cases (one of which required frontal basis reconstruction) and a hemicraniectomy in one case.

**Fig. 2:**
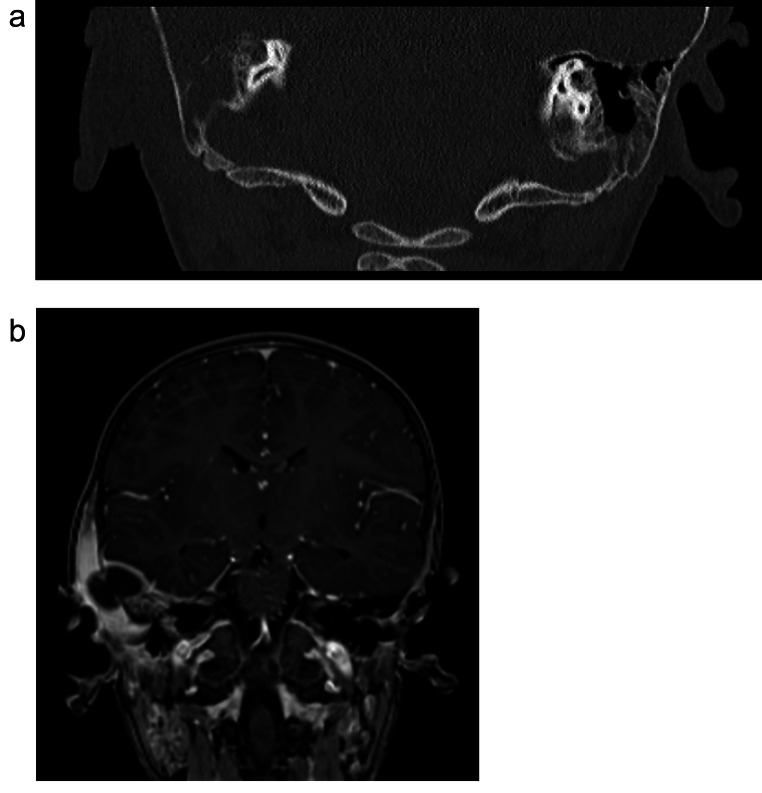
**A**: Coronal CT brain cut bone window showing complete opacification of mastoid air cells, erosion of mastoid air cell bony septum, mastoid cortex destruction and irregularity, and periosteal disruption. **B**: Coronal MR brain with contrast showing signs of pyogenic infection and coalescent mastoiditis, i.e. fluid collection, surrounding contrast enhancement, and periosteal disruption, subperiosteal/subcutaneous abscess

**Fig. 3:**
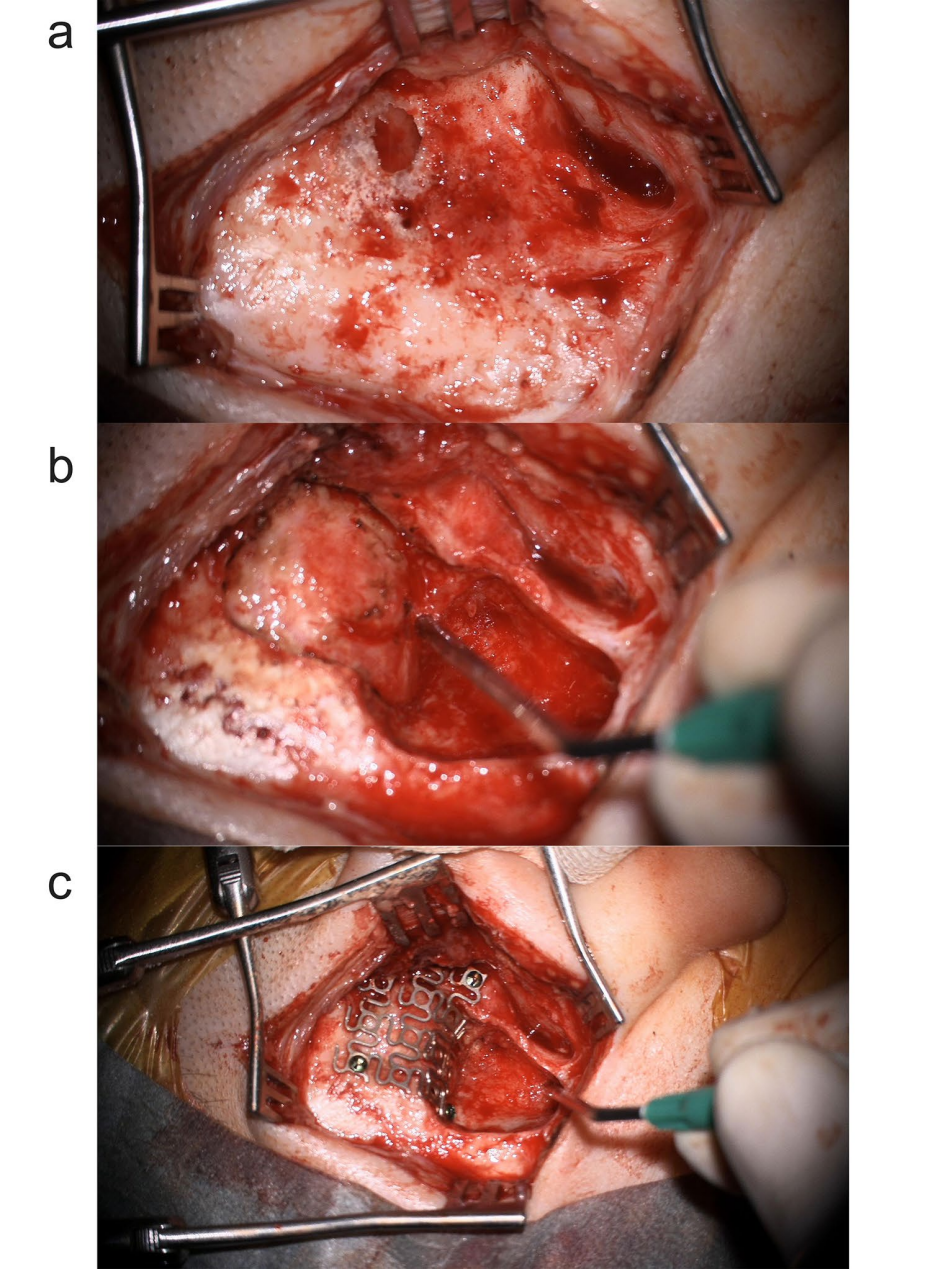
**A**: Intraoperative image after exposure of mastoid process showing erosion of the squamous part of temporal bone. **B**: Intraoperative image after performing full mastoidectomy as well as craniectomy of affected bone of temporal squama and evacuation of epidural abscess. **C**: Intraoperative image showing reconstruction of temporal basis using titanium mesh

In the after-onset of COVID-19 group, the average number of surgeries for neurosurgical procedures was 1.27 and for otorhinolaryngological procedures 1.14. The source of intracranial infection after onset of the COVID-19 pandemic was equally divided between otitis and sinusitis. The otorhinolaryngological surgical interventions, were full mastoidectomies and ventilation tube placements in cases with otogenic origin, and endoscopic sinus surgeries as detailed before in the sinogenic cases. In all 3 otogenic cases, the otorhinolaryngologic and the neurosurgical procedures were performed in a single combined intervention given the erosion of the tegmen mastoid (Figs. 2 and 3). The neurosurgical approaches in the second group comprised a temporal craniotomy in three cases (42.8%), a suboccipital craniotomy in one case (14.3%), a frontal craniotomy with frontal basis reconstruction in two cases (28.7%), and a midline frontal craniotomy in one case (14.3%). In one instance, a second otorhinolaryngological sinus surgery was necessary to extend the surgical window of the frontal sinuses. Additionally, a second neurosurgical operation was required in one case due to residual empyema, along with the placement of a subdural drain.

All surgical approaches and procedures were tailored to each patient’s specific condition and the extent of the intracranial infection, aiming to ensure effective treatment and successful outcomes.

### Antibiotic treatment, microbiological profile, and clinical course

In the pre-COVID-19 group, all three patients had received pretreatment with antibiotics for several days (7, 7 and 6 days, respectively) due to symptoms of sinusitis. In the after-onset of COVID-19 group, one patient with sinusitis had not received any previous antibiotic treatment, while the remaining five had already been treated with antibiotics for varying periods, ranging from 1 to 30 days.

Following the surgery, the patients underwent an empiric regimen of intravenous antibiotics, as detailed in the Table 1. After receiving the microbiological culture and sensitivity report, the therapy was deescalated according to the specific antibiogram in the half of the patients of the after-onset group and in none in the pre-COVID-19 group whenever the bacteria were reported to be sensitive to a single antibiotic. The duration of intravenous antibiotic therapy varied based on the clinical course and radiological findings of the in-patient follow-up MRI examinations. In the pre-COVID-19 group, 66.7% (*N* = 2) of patients continued intravenous antibiotic therapy for 6 weeks, while 33.3% (*N* = 1) received treatment for 12 weeks. In the after-onset of COVID-19 group, the duration of antibiotic therapy was 3 weeks for one patient (14.3%), 4 weeks for three patients (42.9%), and 6 weeks for another two patients (28.7%). No patients received oral antibiotics.

The microorganisms isolated from intraoperative specimens in the first group were Streptococcus intermedius (*N* = 1, 33.3%) and Prevotella loescheii (*N* = 1, 33.3%), while no pathogen could be identified in the specimen from the third patient, most likely due to previous antibiotic treatment with ampicillin and cefotaxime. In the after-onset of COVID-19 group, the microbiological findings indicated the presence of Streptococcus pneumoniae (*N* = 1, 14.3%) due to an otogenic association, Streptococcus intermedius (*N* = 2, 28.7%), Staphylococcus epidermidis (*N* = 1, 14.3%), or a combination of both Streptococcus intermedius and Staphylococcus epidermidis (*N* = 1, 14.3%). Among the patients in the post-COVID-19 onset group with otogenic intracranial infections, two cases (28.7%, *N* = 2) showed the absence of isolated microorganisms in the samples collected. Table 1 shows all microbiological details.

Despite the severity of the disease, all cases showed very good recovery. One patient in the pre-COVID-19 group developed epilepsy and remain showing a mild facial palsy, while in the after-onset of COVID-19 group, one case exhibited residual neurological symptoms, including a mild speech disorder and difficulties in concentrating at school. These successful outcomes emphasize the importance of timely and appropriate interdisciplinary surgical interventions as well as antibiotic treatment in managing these intracranial infections.

## Discussion

### Comparison to other infectiological reports

Throughout the COVID-19 pandemic, changes in the frequency of various infections have been observed in clinical practice [15, 24, 27]. In the field of otorhinolaryngology, studies have reported a reduced number of otogenic and sinogenic infections [10, 17, 19, 23]. Despite the decline in infection cases, recent case reports and clinical studies have identified a worrisome trend of increased severity in sinusitis and otitis media among children, resulting in multiple intracranial complications including extensions of epidural and subdural infections, as well as cerebral venous sinus thrombosis [1, 2, 5, 13, 14, 18, 20, 25, 26]. In our study, we observed a sudden increase in intracranial infections of otogenic origin after the beginning of the pandemic (3 cases versus no cases before the pandemic over the observational period of our study), while those of sinogenic origin remained comparably stable. Interestingly, two cases of mastoiditis in our study after onset of COVID-19 yielded no identifiable pathogens, likely due to the patients’ prior antibiotic treatment. However, the third case of complicated otitis media was caused by Streptococcus pneumoniae, a common pathogen in this type of infection. The pathogens involved in the pathophysiology of sinogenic intracranial infections were as expected, predominantly from the Streptococcus species, with the presence of Staphylococcus epidermidis most probably considered as contamination. Similar to their work, we also noticed an increase of sinus thrombosis as a complication of mastoiditis with or without development of intracranial abscesses, we observed 5 children with such complication, one child needed a VP shunt due to development of increased intracranial pressure and papilledema which did not resolve under medical treatment and serial lumbar punctures.

Hall et al. presented one of the largest series of intracranial empyemas following COVID-19 in 16 children. Their study revealed a four-fold increase in pediatric intracranial empyema cases within the first two years since the onset of the COVID-19 pandemic. Among those cases, 25% of patients had confirmed COVID-19 infections, and of those, 75% exhibited cerebral sinus thromboses, compared to only 25% of non-COVID-19 cases. Similar to our study, 73% of the COVID-19 cases were associated with sinusitis, with Streptococcus intermedius being the most commonly cultured species. In the long-term follow-up, all patients recovered without any limitations [13].

Sutter et al. observed a notable significant rise in cerebral venous sinus thrombosis associated with sinusitis and/or otitis media following the COVID-19 pandemic compared to the pre-COVID-19 era. The majority of cases required anticoagulation therapy, with no reported complications [25]. As mentioned before, same observation was noticed during same period with an increase in patients who developed sinus thrombosis secondary to mastoiditis with or without development of intracranial abscess.

Another study by Khoun et al. reported that pediatric intracranial infections due to rhinosinusitis, otitis media, or mastoiditis increased during the period from March 2020 to March 2022 in 8 pediatric hospitals [17]. During the early COVID-19 pandemic, isolated intracranial abscesses increased by a mean of 100.9%, and sinusitis complicated by intracranial abscess increased by a mean of 76.7% in the participating institutions [17]. However, mastoiditis complicated by intracranial abscess decreased by 116.7% [17]. In our study, we observed a 100% increase in sinogenic and otogenic intracranial infections from one year to three years after the beginning of the pandemic.

Angelo et al. reported at an institutional level an approximately threefold increase in cases of intracranial infections related to sinusitis and otitis media during the Covid pandemic [2]. Interestingly, these reported cases mostly involved infants and children under 12 years old, showing no difference between the patients before and during the COVID-19 pandemic [2]. In contrast, our study revealed that the patients in the after-onset of COVID-19 group were younger than those in the group before the pandemic (9.7 (range 2.2–18.0) vs. 13.3 (range 9.9–16.7) years of age). Notably, Angelo et al. reported a variety of organisms in their study, with Streptococcus constellatus, S. anginosus, S. intermedius, and Parvimonas micra being more prevalent in the COVID-19 cohort [2]. The higher prevalence of the anaerobic Parvimonas in their study may partially explain the discrepancy between their results and those of Khoun, which focused on streptococcal infections [17]. In our study, the microbiological results showed a growth of Streptococcus intermedius in 42.9% (*N* = 3) and pneumoniae in 14.3% (*N* = 1) in the after-onset COVID-19 group, while Staphylococcus epidermidis showed a growth in 28.7% (*N* = 2).

While both our study and recent research have reported a mortality rate of 0%, and all patients recovered without any deficits, the most recent study by Massimi et al. revealed a mortality rate of 2.7%. This study collected 254 cases from 30 centers, indicating a three-fold increase in pediatric patients with sinogenic and otogenic intracranial infections since the onset of COVID-19. Additionally, 95% of the patients required surgical intervention [20].

On the other hand, Kadambari et al. supported a decrease in the prevalence of respiratory diseases during the pandemic and among severe invasive infections, meningitis also showed a significant reduction [15]. If the incidence of otitis in general is thought to have decreased since the beginning of the pandemic, there could possibly be a mechanism causing a more malignant course of the disease associated with intracranial invasiveness. Studies have described high inflammation of the upper respiratory tract mucosa in adults infected with SARS-CoV-2, and a similar mucosal change could be assumed for the pediatric population as well [3].

### Covid-Immunity “Gap”

Since the beginning of the pandemic and the implementation of various protective measures, concerns have been raised about the consequences of social isolation on the immune system in children. Most children had very few social contacts during the pandemic and had to stop visiting preschools, schools, and playgrounds in the early phase of the pandemic. The associated reduced circulation of microbial agents and reduced vaccine uptake probably induced an “immunity debt,” resulting in a growing proportion of susceptible individuals [8]. There have been increases in the incidences of Streptococcus pneumoniae and invasive group A strep (iGAS) infections, likely due to the role of Non-pharmaceutical interventions (NPIs) in reducing viral infections and limiting bacterial superimposed infections [22]. Poor exposure to S. pneumoniae and iGAS due to NPI implementation may have led to a significant immunity debt in relevant population groups, now favoring infection development. The SARS-CoV-2 virus, shows in general a mild course in pediatric populations, but an increased incidence of severe croup, invasive fungal sinus disease, and multi-system inflammatory syndrome (MIS-C) has been noticed in association with the COVID-19 pandemic [7, 22].

### Socioeconomic parameters

While delayed diagnosis and treatment could be a possible explanation for the increased number of cases of complicated otitis and sinusitis, all patients in our study had sought medical attention and received out-patient pretreatment for the primary infection many days before the diagnosis of intracranial invasion. Moreover, they showed no socioeconomic restrictions that could explain a delayed access to medical care facilities.

### Limitations

The findings of this study should be interpreted with caution due to its retrospective design and the small sample size of patients. The documentation of patients’ vaccination history and recent viral infections, including COVID-19, was infrequently recorded in the medical records, which may affect the comprehensive analysis of these factors. Furthermore, the antibody titer of a previous COVID infection was not determined preoperatively in the after-onset COVID group, thus rendering it impossible to make any statement regarding exposure to the Corona virus. Moreover, there is a lack of a comparative group for infection-related surgeries encompassing all identified cases of otitis media and sinusitis infections, which would allow contextualizing the observed increase.

To achieve more robust and generalizable results, multi-center studies involving multiple surgeons and a larger patient population with longer follow-up are warranted. Despite these limitations, this study stands as one of the few comparative analyses exploring the impact of COVID-19 on otogenic and sinogenic intracranial abscesses.

## Conclusion

Since the beginning of the Covid pandemic, concerns have been raised about the consequences of the virus and various protective measures on the immune system of the pediatric population, as well as on the incidence and severity of other infections. In our study, we documented and analyzed a 5-times increase in intracranial infections of sinogenic and especially otogenic origin in our center’s pediatric population, focusing on various demographic, clinical, and microbiological factors. Further research and data from multiple centers are needed to generalize these observations and delve into possible causes and correlations with the Covid pandemic. Understanding these patterns is crucial for better preparedness and management of infectious diseases during such global health emergencies.

## Data Availability

The data presented in this study are available on request from the corresponding author. The data are not publicly available due to contained information compromising privacy necessitating informed consent.
